# Monogalactosyldiacylglycerol and Sulfolipid Synthesis in Microalgae

**DOI:** 10.3390/md18050237

**Published:** 2020-05-01

**Authors:** Gennaro Riccio, Daniele De Luca, Chiara Lauritano

**Affiliations:** 1 Department of Marine Biotechnology, Stazione Zoologica Anton Dohrn, CAP80121 Naples, Italy; gennaro.riccio@szn.it; 2 Department of Humanities, Università degli Studi Suor Orsola Benincasa, CAP80135 Naples, Italy; daniele.deluca088@gmail.com

**Keywords:** microalgae, monogalactosyldiacylglycerol synthase, UDP-sulfoquinovose synthase, sulfoquinovosyltransferase, monogalactosyldiacylglycerols, sulfoquinovosyldiacylglycerols, transcriptome analysis

## Abstract

Microalgae, due to their huge taxonomic and metabolic diversity, have been shown to be a valuable and eco-friendly source of bioactive natural products. The increasing number of genomic and transcriptomic data will give a great boost for the study of metabolic pathways involved in the synthesis of bioactive compounds. In this study, we analyzed the presence of the enzymes involved in the synthesis of monogalactosyldiacylglycerols (MGDGs) and sulfoquinovosyldiacylglycerols (SQDG). Both compounds have important biological properties. MGDGs present both anti-inflammatory and anti-cancer activities while SQDGs present immunostimulatory activities and inhibit the enzyme glutaminyl cyclase, which is involved in Alzheimer’s disease. The Ocean Global Atlas (OGA) database and the Marine Microbial Eukaryotic Transcriptome Sequencing Project (MMETSP) were used to search MGDG synthase (MGD), UDP-sulfoquinovose synthase (SQD1), and sulfoquinovosyltransferase (SQD2) sequences along microalgal taxa. *In silico* 3D prediction analyses for the three enzymes were performed by Phyre2 server, while binding site predictions were performed by the COACH server. The analyzed enzymes are distributed across different taxa, which confirms the importance for microalgae of these two pathways for thylakoid physiology. MGD genes have been found across almost all analyzed taxa and can be separated in two different groups, similarly to terrestrial plant MGD. SQD1 and SQD2 genes are widely distributed along the analyzed taxa in a similar way to MGD genes with some exceptions. For Pinguiophyceae, Raphidophyceae, and Synurophyceae, only sequences coding for MGDG were found. On the contrary, sequences assigned to Ciliophora and Eustigmatophyceae were exclusively corresponding to SQD1 and SQD2. This study reports, for the first time, the presence/absence of these enzymes in available microalgal transcriptomes, which gives new insights on microalgal physiology and possible biotechnological applications for the production of bioactive lipids.

## 1. Introduction

Microalgae are eukaryotic photosynthetic microorganisms that are adapted to live in ecologically different habitats, which results in a wide diversity of species and natural products [1,2] with pharmaceutical [3], nutraceutical [4], and cosmeceutical [5] interests. Microalgae can be cultivated in huge quantities and this advantage overcomes the bottleneck of drug discovery from marine macro-organisms and destructive collection practices. In addition, many studies have focused on optimizing the culturing conditions in order to obtain the metabolites of interest or produce them in large amount. Regarding microalgal molecular resources, various microalgal genomes have been sequenced [6]. However, there are still few genomes available compared to the huge number of existing microalgae [6]. This is due to the fact that microalgae, especially dinoflagellates, can have very large genomes, up to 112 Gbp [7]. On the contrary, several transcriptomes are available for microalgae [6]. Many of these have been sequenced thanks to the Marine Microbial Eukaryote Transcriptome Sequencing Project (MMETSP), the TARA ocean, and the Global Ocean Sampling expeditions, while others are on the way. This will also help discover the enzymes involved in the synthesis of bioactive metabolites and suggest which are the most promising species for genetic engineering manipulations in order to increase the production of specific metabolites.

At the moment, there are only a few studies on metabolic pathways involved in the synthesis of bioactive compounds from microalgae [7,8,9,10,11]. The aim of this paper was to investigate the presence in microalgae of enzymes involved in the synthesis of specific functional lipids with huge interest for pharmaceutical, nutraceutical, and cosmeceutical applications. In particular, microalgae have been found to contain high contents of monogalactosyldiacylglycerols (MGDGs) [12]. MGDGs are compounds of biotechnological interest because they have been investigated for the potential treatment of human pathologies such as cancer (e.g., human pancreatic cancer cell lines) [13] and inflammation [14]. A purified MGDG, extracted from spinach, is a strong DNA polymerase inhibitor and it enhances chemotherapeutic efficiency in human pancreatic cancer cell lines (BxPC-3, MIAPaCa2, and PANC-1) [15]. Regarding marine examples, two MGDGs from the diatom *Phaeodactylum tricornutum* showed *in vitro* pro-apoptotic activity on immortal mouse epithelial cell lines (W2 cells) [16].

Two different MGDGs from the green microalga (Chlorophyta) *Tetraselmis chuii* [17] and four MGDGs from *Nannochloropsis granulata* (Ochrophyta, Eustigmatophyceae) [18] showed anti-inflammatory properties. These MGDGs, extracted from both *T. chuii* and *N. granulata*, were able to reduce NO production and inducible nitric oxide synthase (iNOS) protein levels in lipopolysaccharide (LPS)-stimulated RAW264.7 macrophage cells. In addition, sulfoglycolipids, which constitute the anionic fraction of MGDGs [19], also present interesting biological properties, such as glutaminyl cyclase (QC) inhibitory activity [20] and immuno-stimulatory activity [21]. Sulfolipids extracted from the green microalgae *Tetradesmus lagerheimii* (formerly *Scenedesmus acuminatus*), *Scenedesmus producto-capitatus*, *Pectinodesmus pectinatus* (formerly *Scenedesmus pectinatus*), and *Tetradesmus wisconsinensis* are able to inhibit QC [20] (an enzyme involved in Alzheimer’s disease progression [22]) and, thus, have potential as lead compounds against Alzheimer’s disease. Furthermore, a synthetic sulfolipid derived from *Thalassiosira weissflogii* CCMP1336 (renamed *Conticribra weissflogii*, Bacillariophyta), named β-SQDG18, is a potent vaccine adjuvant [21]. β-SQDG18 is able to trigger a more effective immune response against cancer cells to improve dendritic cell (DC) maturation, to increase CD83-positive DC, and to stimulate the production of pro-inflammatory cytokines (IL-12 and INF-γ) [21].

The aim of the present work was to investigate the presence of genes involved in MGDG and sulfoglycolipid synthesis in microalgae (Bacillariophyta, Cercozoa, Chlorophyta, Chrysophyceae, Coccolithophyceae, Cryptophyta, Dictyochophyceae, Dinophyceae, Euglenophyceae, Eustigmatophyceae, Glaucophyceae, Pavlovophyceae, Pelagophyceae, Pinguiophyceae, Raphidophyceae, Rhodophyta, Synurophyceae, and Xanthophyceae) because little information is available. Our analyses were performed considering all the microalgal transcriptomes and metatranscriptomes available from the MMETSP project and the recent Tara Oceans and Global Ocean sampling expeditions, respectively. 

**Figure 1:**
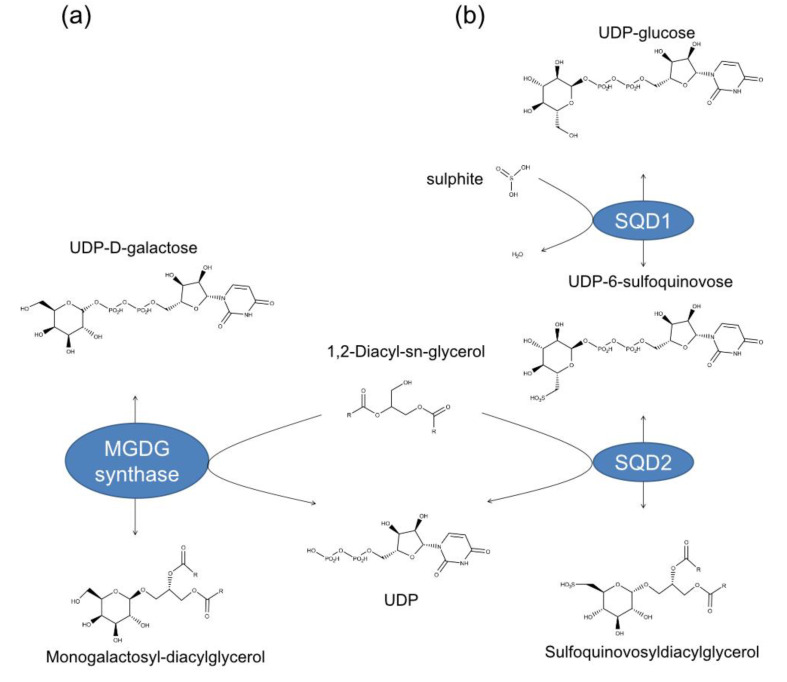
Enzymes responsible for (**a**) monogalactosyldiacylglycerol (i.e., MGDG synthase) and (**b**) sulfoquinovosyldiacylglycerol (i.e., UDP-sulfoquinovose synthase or SQD1 and sulfoquinovosyltransferase or SQD2) biosynthesis.

Galactolipids represent up to 80% of the total lipids of the plastid membranes [23]. They can contain one or two galactose (Gal) molecules, which bond to the glycerol backbone at sn-3 position, and are called, respectively, MGDGs or digalactosyldiacylglycerols (DGDGs). The reaction required for MGDG biosynthesis is catalyzed by a MGDG synthase (MGD), which transfers the Gal moiety from UDP-Gal to diacylglycerol [24] (Figure 1a). MGDG is required in plastids biogenesis and integrity and in photosynthesis [24]. In terrestrial plants, the MGDG synthase is encoded by two types of genes, namely type-A (AtMGD1) and type-B (AtMGD2 and AtMGD3) isoforms, and enzymes are well characterized [25,26,27,28,29,30]. In microalgae, there is no information available yet.

Sulfolipids are constituent of the thylakoids in plant and algal chloroplasts [31]. Sulfoquinovose, which is the building block of sulfolipids, is also the major component of the biological sulfur cycle [32] and it is produced by photosynthetic organisms at a rate of 1010 tons per year [33]. The biosynthesis of these lipids proceeds in two reactions. The first reaction is catalysed by the UDP-sulfoquinovose synthase (SQD1), that assembles the UDP-sulfoquinovose from UDP-glucose and sulphite [34]. The second reaction is catalysed by sulfoquinovosyltransferase (SQD2), which transfers the sulfoquinovose to diacylglycerol [35]. This produces sulfoquinovosyldiacylglycerol (SQDG) (Figure 1b).

Different classes of microalgal-derived compounds have been identified and several have shown specific biological activities, such as anti-cancer [13], anti-inflammatory [14], anti-tuberculosis [36], anti-epilepsy [37], anti-microbial [38], immune-regulatory [39], anti-hypertensive [40], anti-atherosclerosis [40], and anti-osteoporosis [40] activity. The systematic studies of the genes involved in the synthesis of the bioactive compounds of interest are increasing [7,9,10] and can open new perspectives for gene-editing and boost the use of microalgae as a source of new marine natural products.

## 2. Results and Discussion

### 2.1. Identification and Taxonomic Assignation of MGD, SQD1, and SQD2 Homologous Sequences

The BLASTp search conducted in the OGA database returned 1,614 annotated eukaryotic sequences putatively attributable to MGD, 1,422 to SQD1 and 1,340 to SQD2. Of these sequences, the following were unambiguously identified as the genes of interest after Blast2GO analysis: 1154 to MGDG, 817 to SQD1, and 273 to SQD2 (Supplementary Data S1–S3, respectively).

**Figure 2:**
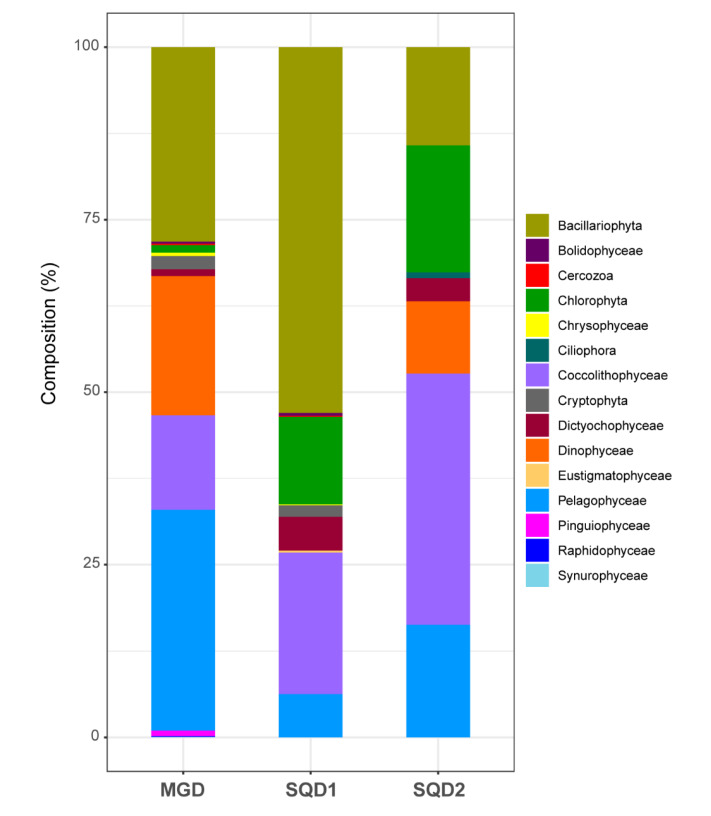
Taxonomic characterization and composition of the analyzed genes. MGD, SQD1, and SQD2 homologs were retrieved from Tara Oceans meta-transcriptomes. Graphical view of taxonomic composition for each gene is reported.

The taxonomic pattern of these sequences, excluding the ones annotated as only as “Eukaryota” and “Stramenopiles” is illustrated in Figure 2. Of the 15 taxonomic divisions considered, 13 were found in the sequences assigned to MGD, 11 to SQD1, and 7 to SQD2 (Figure 2). In this database (OGA), we found homologs for all three genes investigated in the following microalgal taxa: Bacillariophyta, Chlorophyta, Dictyochophyceae, Coccolithophyceae, Pavlovophyceae, and Pelagophyceae. In particular, homolog sequences were particularly abundant in Bacillariophyta, Coccolithophyceae, and Pelagophyceae. For Pinguiophyceae, Raphidophyceae, and Synurophyceae, only sequences coding for MGDG were found. On the contrary, sequences assigned to Ciliophora and Eustigmatophyceae were exclusively corresponding to SQD1 and SQD2. Considering that the results are based on transcriptomic data, they can suggest the absence of a specific gene in certain taxa or simply the analyzed organisms were not expressing the specific gene at the time of sampling and fixing for the RNA-sequencing.

For MMETSP transcriptomes, we retrieved 313 sequences for MGD, 84 for SQD1, and 266 for SQD2 after performing a blastp search. After functional annotation (and removal of sequences considered not valid), 267 sequences were left for MGD, 80 for SQD1, and 121 for SQD2 (Supplementary Data S4–S6, respectively).

We reported in Table 1 the species and the strains in which MGD, SQD1, and SQD2 were found. The analyzed genes were found in 96 different species or strains across 14 different taxonomic categories. All the three genes were found in the reported taxa, with the exception of the two species of Chrysophyceae (*Dinobryon* sp. UTEXLB2267 and *Paraphysomonas imperforata* PA2, Supplementary Data S7). In addition, considering that the results are based on transcriptomic data, they can be interpreted as either the absence of that specific gene or lack of its expression in that particular condition.

### 2.2. Final Dataset and Phylogenetic Inferences

The final alignments consisted of 649 sequences for MGD, 521 for SQD1, and 244 for SQD2 (Supplementary Data S8–S10, respectively), from both the OGA and MMETSP database. The length of each dataset after trimming of poorly aligned regions was as follows: 377 bp for MGD, 198 bp for SQD1, and 295 bp for SQD2.

**Figure 3:**
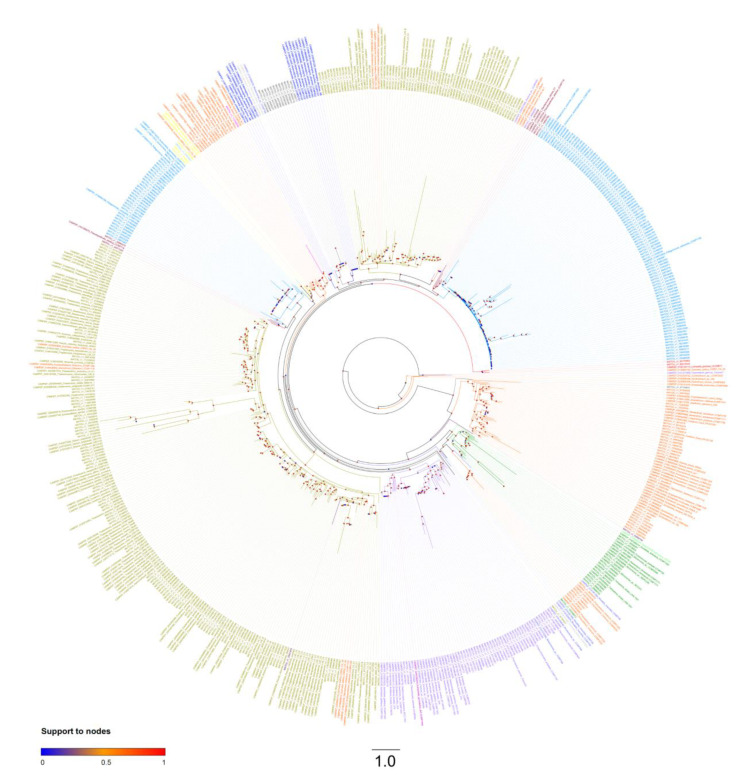
MGD unrooted phylogenetic tree. Colored circles at the base of each node refer to branch support after aLRT SH-like test. Colors of taxa refer to taxonomic groups as in Figure 2 and Table 1.

The MGD phylogenetic tree showed that most of the taxa here investigated contained paralog copies (homolog copies resulting from duplication events) of monogalactosyldiacylglycerol synthase gene (Figure 3). These paralogs generally occurred in two copies, resulting in two distinct and highly supported clades for most taxa (red circles, Figure 3, Supplementary File S1). A few taxa (e.g., Coccolithophyceae, Cryptophyta, and Xantophyceae) presented only one MGD copy (and, therefore, a single group of sequences), while others (e.g., Dinophyceae) presented several MGD paralogs (Figure 3 or Supplementary File S1).

**Figure 4:**
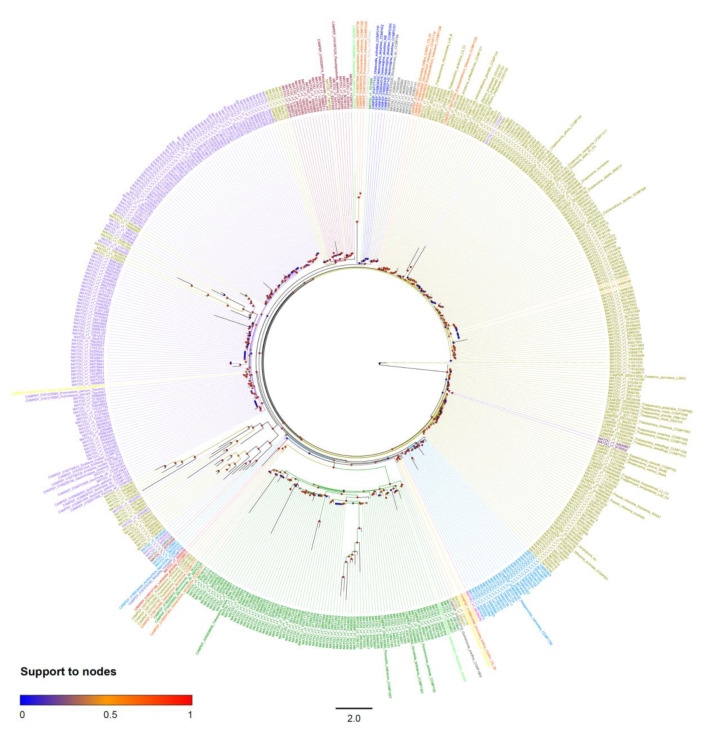
SQD1 unrooted the phylogenetic tree. Colored circles at the base of each node refer to branch support after an aLRT SH-like test. Colors of taxa refer to taxonomic groups in Figure 2 and Table 1.

For the SQD1 gene, the phylogenetic tree showed that all the sequences belonging to the same taxon formed a highly supported monophyletic group in most species (Figure 4, Supplementary File S2). Among the exceptions, there are the diatoms (Bacillariphyta) where, beside a clade containing the most diatom sequences retrieved from OGA and MMETSP, there are a few others interspersed across the tree (Figure 4). One of these only contains sequences of *Thalassiosira rotula* (synonym of *T. gravida*) strain CCMP1093 from MMETSP. The others contain small groups of sequences from OGA (Figure 4, Supplementary File S2). Similarly, SQD1 dinoflagellates (Dinophyceae) homologs do not form a monophyletic group in the tree. This could be due to the high complexity of the plastid evolution in Dinophyceae. Dinophyceae acquired plastid with four different endosymbiotic events. [41]. In a secondary endosymbiotic event, Dinophyceae acquired plastid by serial endosymbiosis with green microalgae (Chlorophyta) [42]. In the tertiary endosymbiotic event, Dinophyceae acquired tertiary plastids by endosymbiosis with Cryptophyceae-Cryptomonads, Haptophyta, and Bacillariphyta [41].

**Figure 5:**
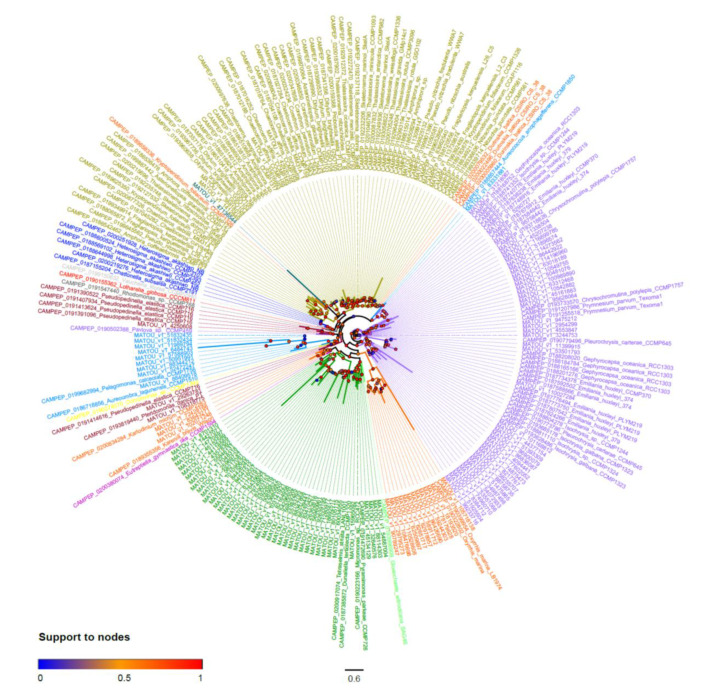
SQD2 unrooted phylogenetic tree. Colored circles at the base of each node refer to branch support after the aLRT SH-like test. Colors of taxa refer to taxonomic groups in Figure 2 and Table 1.

For the SQD2 gene, all the sequences from the same taxonomic group formed monophyletic groups except for the Dinophyceae (Figure 5, Supplementary File S3). All the dinoflagellate sequences from OGA and MMETSP were interspersed across the tree with high support, close to Bacillariophyta, Chlorophyta, Dictyocophyceae, and Coccolithophyceae.

### 2.3. Structural Details of MDGs, SQD1, and SQD2 from Thalassiosira Weissflogii CCMP1336 (Conticriba weissflogii)

**Figure 6:**
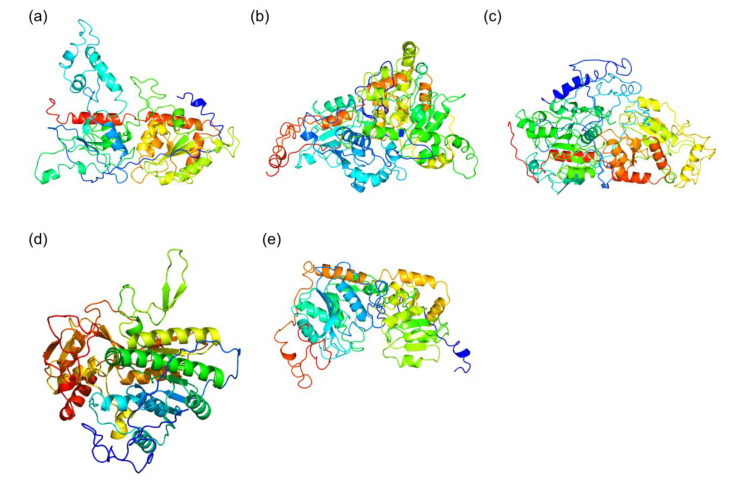
*In silico* model generated by Phyre2 for: (**a**) MGD CAMPEP_0193073380, (**b**) MGD CAMPEP_0193064960, (**c**) MGD CAMPEP_0193062160, (**d**) SQD1 CAMPEP_0193062736, and (**e**) SQD2 CAMPEP_0193058822.

We built *in silico* models for MGDs, SQD1, and SQD2 proteins from the diatom *T. weissflogii* CCMP1336 (synonym of *Conticribra weissflogii*). *T. weissflogii* (*C. weissflogii*) CCMP1336 has been selected because it is known to have immunostimulatory activity, and both galactolipids and sulfolipids have been found to play a key role in this bioactivity [39,43]. 3D *in silico* models (Figure 6) were generated using the amino acid sequences of MGD (CAMPEP_0193073380, CAMPEP_0193064960 and CAMPEP_0193062160) (Figure 6a–c), SQD1 (CAMPEP_0193062736) (Figure 6d), and SQD2 (CAMPEP_0193058822) (Figure 6e), obtained from the MMETSP database. Phyre2 results were summarized in Table 2. The analyses pointed out the homology between *T. weissflogii* (*C. weissflogii*) and *Arabidopsis thaliana* proteins. Structural prediction of MGDs (CAMPEP_0193073380, Supplementary Data S11, CAMPEP_0193064960 Supplementary Data S12, and CAMPEP_0193062160, Supplementary Data S13) found high similarity with the 3D structure of MGD1 from *A. thaliana*. The percentage of identity between *T. weissflogii* (*C. weissflogii*) and *A. thaliana* MGD1 is higher than 40%. Similar results were obtained for structural prediction of SQD1 from *T. weissflogii* (*C. weissflogii*) (Supplementary Data S14) with 45% identity with the 3D structure of SQD1 from *A. thaliana*.

Structural prediction for SQD2 (Supplementary Data S15) found a similarity with the 3D structure of sucrose synthase-1 (SUS1) from *A. thaliana*. The percentage of identity between SQD2 and SUS1 is 18%, which is lower than the percent of identity of SQD1 and MDGs. SUS1 from *A. thaliana* catalyzed a reversible sucrose synthesis. It transfers glucose moiety from UDP to fructose and it has been found to form a complex with both UDP–glucose and UDP–fructose [44]. These findings may indicate the presence of a low specificity ligand-binding site in the SQD2 enzyme.

**Figure 7:**
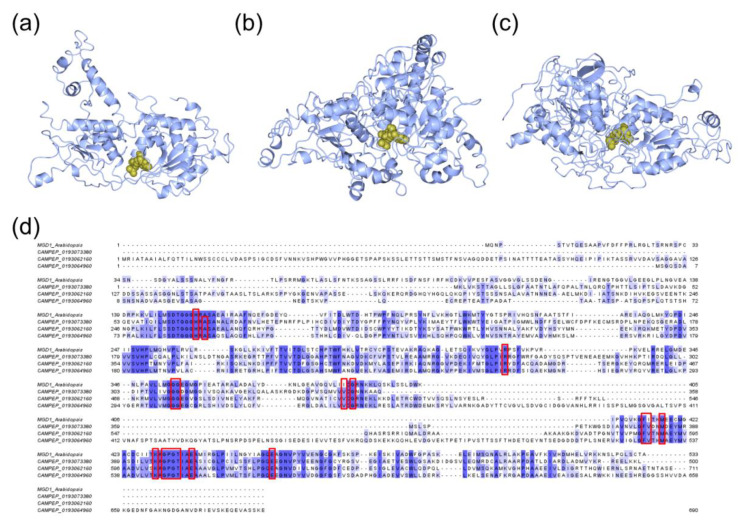
Protein-ligand binding site prediction by the COACH server for MDGs from *Thalassiosira* (*Conticribra*) *weissflogi* CCMP1336. Prediction of binding sites for the three MGDs: (**a**) MGD CAMPEP_0193073380–UDP complex, (**b**) MGD CAMPEP_0193064960–UDP complex, and (**c**) MGD CAMPEP_0193062160–UDP complex. (**d**) Among the Clustal Omega result of MDG amino acid sequences, the red boxes indicated the conserved amino acid residues involved in binding-pockets.

In order to evaluate whether predicted structures preserved the functional active sites of MGDs, SQD1, and SQD2, they were also analyzed by using the COACH server. MGD analysis was performed for all the structures obtained from Phyre2. The analysis pointed out the presence of a UDP binding pocket for the three MGDs. MGD CAMPEP_0193073380 (Figure 7a) binding site prediction had a C-score 0.32 and the presence of 17 residues in the binding site (i.e., H69, A71, R263, G312, G313, V350, G352, F379, V380, M383, Y396, G398, P399, G400, T401, E40, and E422). MGD CAMPEP_0193064960 (Figure 7b) binding site prediction had a low C-score value (0.18) and 17 amino acid residues were involved in the binding site (i.e., H89, A91, R260, G305, G306, V335, G337, F528, V529, M532, K545, G547, P548, G549, T550, E553, and E571). MGD CAMPEP_0193062160 (Figure 7c) binding site prediction had a low C-score (0.18) and 17 residues involved (i.e. H262, A264, R434, G477, G478, V507, G509, F586, V587, M590, K603, G605, P606, G607, T608, E611, and E629). COACH prediction analysis highlighted the same 17 amino acid residues involved in ligand-binding site of the three MDGs. Moreover, clustal omega analysis including MGD1 from *A. thaliana* indicated that these 17 residues were located in highly conserved domains (Figure 7d) and corresponded to the active site of MGD1 from *A. thaliana* [45] with the exception of the alanine residue.

**Figure 8:**
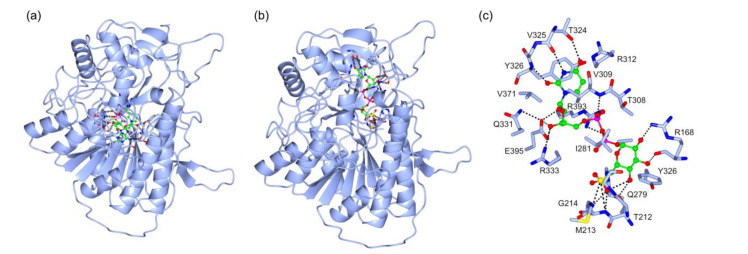
Protein-ligand binding site prediction by COACH server of SQD1 from *Thalassiosira* (*Conticribra*) *weissflogii* CCMP1336. (**a**) Prediction of binging site of the complex SQD1–NAD. (**b**) Prediction of binding site of the complex SQD1–USQ. (**c**) Structure of USQ binding-pocket and the specific interaction between USQ and highlighted amino acid residues of SQD1.

SQD1 analysis indicated the presence of a NAD-binding pocket (Figure 8a) with a C-score of 0.85. The following residues were reported to be involved in the NAD-binding pocket structure: G74, D76, G77, F78, C79, D98, N99, S101, R102, L140, D141, V142, F164, A165, E166, R168, A169, N186, L210, G211, T212, Y252, K256, Q279, G280, I281, V282, and Y306. The presence of a UDP–6–sulfoquinovose (USQ) binding pocket (Figure 8b) was predicted with a C-score of 0.27. The residues involved in USQ-binding pocket were R168, A170, T212, M213, G214, Y252, H253, Q279, G280, I281, T308, V309, R312, T324, Y326, Q331, R333, V371, R393, and E395. Most of them were involved in USQ binding (Figure 8c). The ligand binding pocket structure prediction, such as the predicted structure, was in line with the structure and function of SQD1 from *A. thaliana* [34,46].

**Figure 9:**
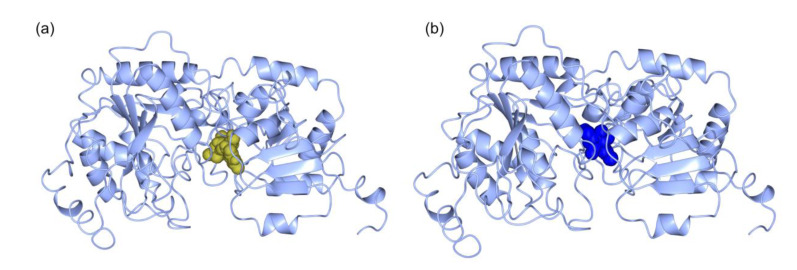
Protein-ligand binding site prediction by COACH server of SQD2 from *Thalassiosira* (*Conticribra*) *weissflogii* CCMP1336. (**a**) Prediction of binding site of the complex SQD2–UDP. (**b**) prediction of binding site of the complex SQD2–NAG.

SQD2 analysis indicated the presence of a UDP-biding pocket (Figure 9a) (C-score 0.28), which involved the residues S82, G83, N86, F256, V282, G283, R284, K289, E309, Q332, L333, L338, E355, G358, F359, V360, and E363. Moreover, COACH analyses pointed out the presence of a N-Acetylglucosamine (NAG)–binding pocket (Figure 9b) (C-score 0.33) that involved the residues G83, Y84, R87, H190, T191, K249, S354, E355, T356, L357, and G358. These data confirmed the presence of a low specificity ligand binding pocket, and suggest that SQD2 from diatoms could be involved in the synthesis of other diacylglycerols such as glucuronosyldiacylglycerol (GlcADG) in *Arabidopsis* [47] or flavonoid glycosylation in *Oryza sativa* [48,49].

## 3. Materials and Methods

### 3.1. Identification of MGD, SQD1, and SQD2 Homologous Sequences

Since our primary interest was to ascertain the occurrence of MGD, SQD1, and SQD2 in microalgae, we used the sequences of MGD (accession number XP002181685), SQD1 (accession number XP002185968), and SQD2 (accession number XP002185276) from the diatom *Phaeodactylum tricornutum* as queries for a BLAST [50] search against the Ocean Global Atlas (OGA, [51]) database (http://tara-oceans.mio.osupytheas.fr/oceangene-atlas/), and several protist transcriptomes from the Marine Microbial Eukaryotic Transcriptome Sequencing Project (MMETSP, [52]) available at https://zenodo.org/record/12125852585. The OGA database contains a collection of more than 116 million eukaryote and 40 million prokaryotic genes gathered during the *Tara* Oceans [53,54] and the Global Ocean Sampling [55] expeditions. Instead, MMETSP contains the transcriptomes of some of the most abundant and ecologically significant microbial eukaryotes in the oceans. To retrieve homologs, we used the blastp algorithm against the metagenome/metatranscriptome and transcriptome databases contained in OGA and the MMETSP, setting the expect threshold to 1E-10. For OGA, after ascertaining that the sequences obtained from metagenomes and metatranscriptomes were identical, we only used one dataset (metatranscriptomes) for further analyses. Since a sequence-based homology search could have recovered different genes with similar functions, we used Blast2GO [56] to obtain a functional annotation of the homologs retrieved. We used the default settings (i.e., blastx program, using the nr BLAST database and with a BLAST expectation value of 1.0E-3) for the analysis. We considered valid Blast2GO annotations containing the following names: 1,2-diacylglycerol 3-beta-galactosyltransferase and monogalactosyldiacylglycerol synthase for MGD, sulfolipid biosynthesis protein, sulfoquinovosyldiacylglycerol synthesis protein, UDP sulfoquinovose synthase and uridine 5′-diphosphate-sulfoquinovose synthase for SQD1, sulfoquinovosyl transferase SQD2, sulfoquinovosyldiacylglycerol 2, and UDP-sulfoquinovose: DAG sulfoquinovosyltransferase for SQD2. All the sequences identified as only “predicted or hypothetical protein” without specification were *a priori* discarded.

### 3.2. Taxonomic Overview

All the sequences from OGA that passed the Blast2GO analysis with the criterions specified above were annotated using the annotation file generated during homolog retrieval. We removed all the sequences without a taxonomic annotation, annotated only as “Eukaryota” and, whenever applicable, with a generic annotation that did not allow to discriminate such sequences from others of lower taxonomic rank (e.g., “Stramenopiles”). All the taxa in which the MGD, SQD1, and SQD2 genes were found and were organized into a table. The abundance of such sequences were plotted as histograms using the R [57] working packages *scales* [58] and *ggplot2* [59]. For the homologs retrieved from the MMETSP transcriptomes, we generated a table illustrating the species surveyed and the genes of interest that were found in each of them.

### 3.3. Sequence Alignment and Phylogenetic Inference

The sequences of each gene from OGA and MMETSP that passed Blast2GO annotation and with a length of at least 200 aa were aligned using COBALT [60] available at https://www.ncbi.nlm.nih.gov/tools/cobalt/). Unlike other common software used for protein alignments (e.g., ClustalW, MAFFT, MUSCLE, ProbCons) that only use sequence information, COBALT also integrates the information of protein-motif regular expressions (PROSITE database) and of conserved protein domains (NCBI CDD database). In doing so, COBALT has a better chance of producing a biologically meaningful multiple alignment compared to tools that do not utilize this information 60]. Poorly aligned regions from each alignment were removed with trimAl v1.2 [61] in order to increase the quality of subsequent phylogenetic analyses. We used the *automated1* option to find the most appropriate mode to trim the alignments (use of gaps or similarity scores) depending on the alignment characteristics.

In order to infer reliable phylogenetic trees, all the sequences that, after trimming, were shorter than one quarter (around 60–70 aa) of the final length of the alignment were removed, unless longer sequences were not available for that particular taxon. Maximum likelihood phylogenetic trees were inferred using PhyML [62] using the LG substitution model [63], which turned out to be the best evolution model for the three genes investigated according to the Akaike Information Criterion implemented in SMS [64]. Support to nodes was calculated using the Shimodaira-Hasegawa-like (aLRT SH-like) procedure [65]. Trees were visualised and graphically edited in FigTree v1.4.3 (http://tree.bio.ed.ac.uk/software/figtree/).

### 3.4. In Silico Protein Model

The three-dimensional (3D) *in silico* models of MGD, SQD1, and SQD2 proteins from *Thalassiosira weissflogii* CCMP1336 (currently regarded as synonym of *Conticribra weissflogii*, Bacillariophyta) were generated using Phyre2 server (http://www.sbg.bio.ic.ac.uk/~phyre2/html/page.cgi?id=index) [66]. Protein ligand-binding site predictions were performed using COACH analyses from the Zhang Lab server. COACH generated ligand-binding site predictions and a confidence score (C-score) of the prediction. The C-score ranged from 0 to 1. A higher score indicates a reliable prediction (https://zhanglab.ccmb.med.umich.edu/COACH/) [67,68]. Pictures were obtained using CCP4MG, version 2.10.11 obtained by http://www.ccp4.ac.uk/MG/download/ [69].

## 4. Conclusions

This study analyzes the presence of MGD, SQD1, and SQD2 in microalgae and gives a broad overview of their presence among different microalgal classes. It has been shown that, within each taxa, some species do not express all the sequences, which suggests the absence of a specific gene. Despite the fact that several species have been found to express MGDs, SQD1, and SQD2 (Table 1), only a relatively small number of active microalgae have been studied and reported to possess anti-inflammatory/immunomodulatory activities, including the diatoms *Attheya longicornis*, *Cylindrotheca closterium*, *Trieres mobiliensis* (formerly *Odontella mobiliensis*), *Phaeodactylum tricornutum*, *Porosira glacialis*, *Pseudo-nitzschia pseudodelicatissima,* and *Thalassiosira* (*Conticribra*) *weissflogii*, and the flagellates *Amphidinium carterae* (Dinophyceae), *Edaphochlamys debaryana* (formerly *Chlamydomonas debaryana*), *Chloroidium saccharophilum* (formerly *Chlorella ovalis*), *Dunaliella salina* (formerly *Dunaliella bardawil*) (Chlorophyceae), *Nannochloropsis granulata*, *Nannochloropsis oculata* (Eustigmatophyceae), *Diacronema lutheri* (formerly *Pavlova lutheri*, Pavlovophyceae), *Tetraselmis chuii,* and *Tetraselmis suecica* (Chlorophyta, Chlorodendrophyceae) [39]. Our study can direct and accelerate the discovery of new bioactive species and give new insights on enzyme discovery from microalgae with biotechnological applications.

3D *in silico* prediction analyses indicated that the enzymes, maintaining conserved domains, could be effectively involved in the synthesis of compounds with known anticancer and immune-modulatory activities, such as MGDGs and SQDGs. This approach can give preliminary information for the selection of specific microalgal species for drug discovery and for genetic engineering approaches in order to produce huge amounts of bioactive compounds of pharmaceutical interest.

## Figures and Tables

**Table 1:** Species and strains from MMETSP transcriptomes in which MGD, SQD1, and SQD2 homologs were found. Colors refer to taxonomic ranks as in Figure 2. P = presence of validated gene homologs.

Color Legend	Taxonomic Group	Species/Strain Surveyed	Accepted Synonym	MGD	SQD1	SQD2
	Dinophyceae	*Alexandrium monilatum* CCMP3105		P	P	P
	Dinophyceae	*Alexandrium tamarense* CCMP1771		P	P	
	Dinophyceae	*Amphidinium carterae* CCMP1314		P		
	Bacillariophyta	*Amphiprora* sp.		P	P	P
	Bacillariophyta	*Amphora coffeaeformis* CCMP127	*Halamphora coffeiformis*	P	P	P
	Bacillariophyta	*Asterionellopsis glacialis* CCMP134		P	P	P
	Pelagophyceae	*Aureococcus anophagefferens* CCMP1850		P		P
	Pelagophyceae	*Aureoumbra lagunensis* CCMP1510		P	P	P
	Dinophyceae	*Azadinium spinosum* 3D9		P		
	Bacillariophyta	*Ceratium fusus* PA161109	*Tripos fusus*	P		
	Bacillariophyta	*Chaetoceros affinis* CCMP159		P	P	P
	Bacillariophyta	*Chaetoceros curvisetus*		P	P	P
	Bacillariophyta	*Chaetoceros debilis* MM31A 1		P	P	P
	Bacillariophyta	*Chaetoceros neogracile* CCMP1317		P	P	P
	Raphidophyceae	*Chattonella subsalsa* CCMP2191		P	P	P
	Coccolithophyceae	*Chrysochromulina polylepis* CCMP1757	*Prymnesium polylepis*	P	P	P
	Bacillariophyta	*Corethron pennatum* L29A3		P	P	P
	Dinophyceae	*Crypthecodinium cohnii* Seligo		P		
	Bacillariophyta	*Ditylum brightwellii* GSO103		P	P	P
	Bacillariophyta	*Ditylum brightwellii* GSO104		P	P	P
	Bacillariophyta	*Ditylum brightwellii* GSO105		P	P	P
	Chlorophyta	*Dunaliella tertiolecta* CCMP1320		P	P	P
	Dinophyceae	*Durinskia baltica* CSIRO CS 38	*Durinskia dybowskii*	P	P	P
	Coccolithophyceae	*Emiliania huxleyi* 374		P		P
	Coccolithophyceae	*Emiliania huxleyi* 379		P	P	P
	Coccolithophyceae	*Emiliania huxleyi* CCMP370		P	P	P
	Coccolithophyceae	*Emiliania huxleyi* PLYM219		P	P	P
	Euglenophyceae	*Eutreptiella gymnastica* like CCMP1594		P		P
	Bacillariophyta	*Extubocellulus spinifer* CCMP396		P	P	
	Bacillariophyta	*Fragilariopsis kerguelensis* L26 C5		P	P	P
	Bacillariophyta	*Fragilariopsis kerguelensis* L2 C3		P	P	P
	Coccolithophyceae	*Gephyrocapsa oceanica* RCC1303		P	P	P
	Dinophyceae	*Glenodinium foliaceum* CCAP1116 3	*Kryptoperidinium foliaceum*	P	P	P
	Glaucophyceae	*Gloeochaete wittrockiana* SAG46 84		P	P	P
	Cryptophyta	*Goniomonas pacifica* CCMP1869			P	
	Raphidophyceae	*Heterosigma akashiwo* CCMP2393		P	P	P
	Raphidophyceae	*Heterosigma akashiwo* CCMP3107		P	P	P
	Raphidophyceae	*Heterosigma akashiwo* CCMP452		P	P	P
	Raphidophyceae	*Heterosigma akashiwo* NB		P	P	P
	Coccolithophyceae	*Isochrysis galbana* CCMP1323		P	P	P
	Coccolithophyceae	*Isochrysis* sp. CCMP1244		P	P	P
	Coccolithophyceae	*Isochrysis* sp. CCMP1324		P	P	P
	Dinophyceae	*Karenia brevis* CCMP2229		P		
	Dinophyceae	*Karenia brevis* SP1		P		
	Dinophyceae	*Karenia brevis* SP3		P		
	Dinophyceae	*Karenia brevis* Wilson		P		P
	Dinophyceae	*Karlodinium micrum* CCMP2283	*Karlodinium veneficum*	P		P
	Dinophyceae	*Kryptoperidinium foliaceum* CCMP1326		P	P	P
	Dinophyceae	*Lingulodinium polyedra* CCMP1738		P	P	
	Cercozoa	*Lotharella globosa* CCCM811		P	P	P
	Chlorophyta	*Micromonas* sp. CCMP2099		P		
	Chlorophyta	*Micromonas* sp. NEPCC29		P	P	
	Chlorophyta	*Micromonas* sp. RCC472		P	P	P
	Bacillariophyta	*Nitzschia punctata* CCMP561	*Tryblionella punctata*	P	P	P
	Chrysophyceae	*Ochromonas* sp. CCMP1393		P	P	P
	Dinophyceae	*Oxyrrhis marina*		P		P
	Dinophyceae	*Oxyrrhis marina* LB1974				P
	Pavlovophyceae	*Pavlova* sp. CCMP459		P	P	P
	Pelagophyceae	*Pelagococcus subviridis* CCMP1429		P		
	Pelagophyceae	*Pelagomonas calceolata* CCMP1756		P	P	P
	Dinophyceae	*Peridinium aciculiferum* PAER 2	*Apocalathium aciculiferum*	P		
	Chlorophyta	*Picocystis salinarum* CCMP1897			P	
	Coccolithophyceae	*Pleurochrysis carterae* CCMP645	*Chrysotila carterae*	P		P
	Bacillariophyta	*Proboscia alata* PI D3		P	P	P
	Dinophyceae	*Prorocentrum minimum* CCMP1329	*Prorocentrum cordatum*	P	P	
	Dinophyceae	*Prorocentrum minimum* CCMP2233	*Prorocentrum cordatum*	P	P	
	Coccolithophyceae	*Prymnesium parvum* Texoma1		P	P	P
	Bacillariophyta	*Pseudo-nitzschia australis* 10249 10 AB		P	P	P
	Bacillariophyta	*Pseudo-nitzschia fradulenta* WWA7		P	P	P
	Dictyochophyceae	*Pseudopedinella elastica* CCMP716		P	P	P
	Dictyochophyceae	*Pteridomonas danica* PT		P	P	P
	Chlorophyta	*Pyramimonas parkeae* CCMP726		P	P	P
	Rhodophyta	*Rhodella maculata* CCMP736	*Rhodella violacea*	P	P	
	Cryptophyta	*Rhodomonas* sp. CCMP768		P	P	P
	Dinophyceae	*Scrippsiella hangoei* SHTV5	*Apocalathium malmogiense*	P		
	Dinophyceae	*Scrippsiella hangoei* like SHHI 4		P		
	Dinophyceae	*Scrippsiella trochoidea* CCMP3099		P		
	Bacillariophyta	*Skeletonema dohrnii* SkelB		P	P	P
	Bacillariophyta	*Skeletonema marinoi* SkelA		P	P	P
	Bacillariophyta	*Skeletonema menzelii* CCMP793		P	P	P
	Dinophyceae	*Symbiodinium* sp. C1		P		
	Dinophyceae	*Symbiodinium* sp. CCMP2430		P		
	Dinophyceae	*Symbiodinium* sp. Mp		P		
	Chlorophyta	*Tetraselmis striata* LANL1001		P	P	P
	Bacillariophyta	*Thalassionema nitzschioides* L26 B		P		P
	Bacillariophyta	*Thalassiosira antarctica* CCMP982		P	P	P
	Bacillariophyta	*Thalassiosira gravida* GMp14c1		P	P	P
	Bacillariophyta	*Thalassiosira miniscula* CCMP1093		P	P	P
	Bacillariophyta	*Thalassiosira oceanica* CCMP1005		P	P	P
	Bacillariophyta	*Thalassiosira rotula* CCMP3096	*Thalassiosira gravida*	P	P	P
	Bacillariophyta	*Thalassiosira rotula* GSO102	*Thalassiosira gravida*	P	P	P
	Bacillariophyta	*Thalassiosira weissflogii* CCMP1010	*Conticribra weissflogii*	P	P	
	Bacillariophyta	*Thalassiosira weissflogii* CCMP1336	*Conticribra weissflogii*	P	P	P
	Bacillariophyta	*Thalassiothrix antarctica* L6 D1		P	P	
	Xanthophyceae	*Vaucheria litorea* CCMP2940		P	P	P

**Table 2:** Report of Phyre2 analysis. We report the template (protein of known structure used for the prediction analysis) and its protein data bank (PDB) code, confidence (probability that the sequence and template are homologous), and percent id (percent of identity).

*Thalassiosira* (*Conticribra*) *weissflogii* CCMP1336 Proteins	Template (PDB Code)	Confidence		% id
MGD CAMPEP 0193073380	MGD1 from *A. thaliana* (4X1T)		100	46
MGD CAMPEP 0193064960	MGD1 from *A. thaliana* (4X1T)		100	47
MGD CAMPEP 0193062160	MGD1 from *A. thaliana* (4X1T)		100	40
SQD1 CAMPEP_0193062736	SQD1 from *A. thaliana* (1I24)		100	45
SQD2 CAMPEP_0193058822	SUS1 from *A. thaliana* (3S29)		100	18

## Supplementary Materials

The following are available online at https://www.mdpi.com/1660-3397/18/5/237/s1. **Data S1**: Blast2GO analysis of MGD genes. BLASTp search conducted in the OGA database highlighted 1614 annotated eukaryotic sequences putatively attributable to MGD. Of these sequences, 1154 were identified as the gene of interest. **Data S2**: Blast2GO analysis of SQD1 genes. BLASTp search conducted in the OGA database highlighted 1422 annotated eukaryotic sequences putatively attributable to SQD1. Of these sequences, 817 were identified as the gene as the gene of interest. **Data S3**: Blast2GO analysis of SQD2 genes. BLASTp search conducted in the OGA database highlighted 1340 annotated eukaryotic sequences putatively attributable to SQD2. Of these sequences, 273 were identified as the gene of interest. **Data S4**: MGD amino acid sequences from MMETSP transcriptomes. After functional annotation, 267 sequences were left for MGD. **Data S5**: SQD1 amino acid sequences from MMETSP transcriptomes. After functional annotation, 84 sequences were left for MGD. **Data S6**: SQD2 amino acid sequences from MMETSP transcriptomes. After functional annotation, 121 sequences were left for SQD2. **Data S7:** Original data for Table 1. All the species and the strains were analyzed from MMETSP. **Data S8**: Alignments for MGD amino acid sequences. The alignments were performed using 649 amino acid sequences for MGD. **Data S9**: Alignments for SQD1 amino acid sequences. The alignments were performed using 521 amino acid sequences for SQD1. **Data S10:** Alignments for SQD2 amino acid sequences. The alignments were performed using 295 amino acid sequences for SQD2. **Data S11**: PDB file of MGD CAMPEP_0193073380 predicted structure. *In silico* model generated by Phyre2 for MGD CAMPEP_0193073380. **Data S12**: PDB file of MGD CAMPEP_0193064960 predicted structure. *In silico* model generated by Phyre2 for MGD CAMPEP_0193064960. **Data S13:** PDB file of MGD CAMPEP_0193062160 predicted structure. *In silico* model generated by Phyre2 for MGD CAMPEP_0193062160. **Data S14:** PDB file of SQD1 CAMPEP_0193062736 predicted structure. *In silico* model generated by Phyre2 for SQD1 CAMPEP_0193062736. Additional file 15: Data S15: PDB File of SQD2 CAMPEP_0193058822 predicted structure. *In silico* model generated by Phyre2 for SQD2 CAMPEP_0193058822. **File S1**: PDF file of MGD unrooted phylogenetic tree. Colored circles at the base of each node refer to branch support after aLRT SH-like test. Colors of taxa refer to taxonomic groups as in Table 1. **File S2**: PDF file of SQD1 unrooted phylogenetic tree. Colored circles at the base of each node refer to branch support after aLRT SH-like test. Colors of taxa refer to taxonomic groups in Table 1. **File S3**: PDF file of SQD2 unrooted phylogenetic tree. Colored circles at the base of each node refer to branch support after the aLRT SH-like test. Colors of taxa refer to taxonomic groups as in Table 1.
