# Supplementary figures and images for: Monogalactosyldiacylglycerol and Sulfolipid Synthesis in Microalgae
Source: Mar Drugs. 2020 May 1;18(5):237. doi: 10.3390/md18050237 (PMC7281551; doi:10.3390/md18050237)

Support to nodes

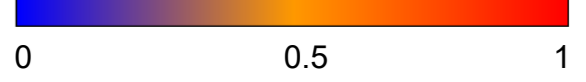

Supplement: Supplementary file 1 [file marinedrugs-18-00237-s001.zip › S1 File.pdf]

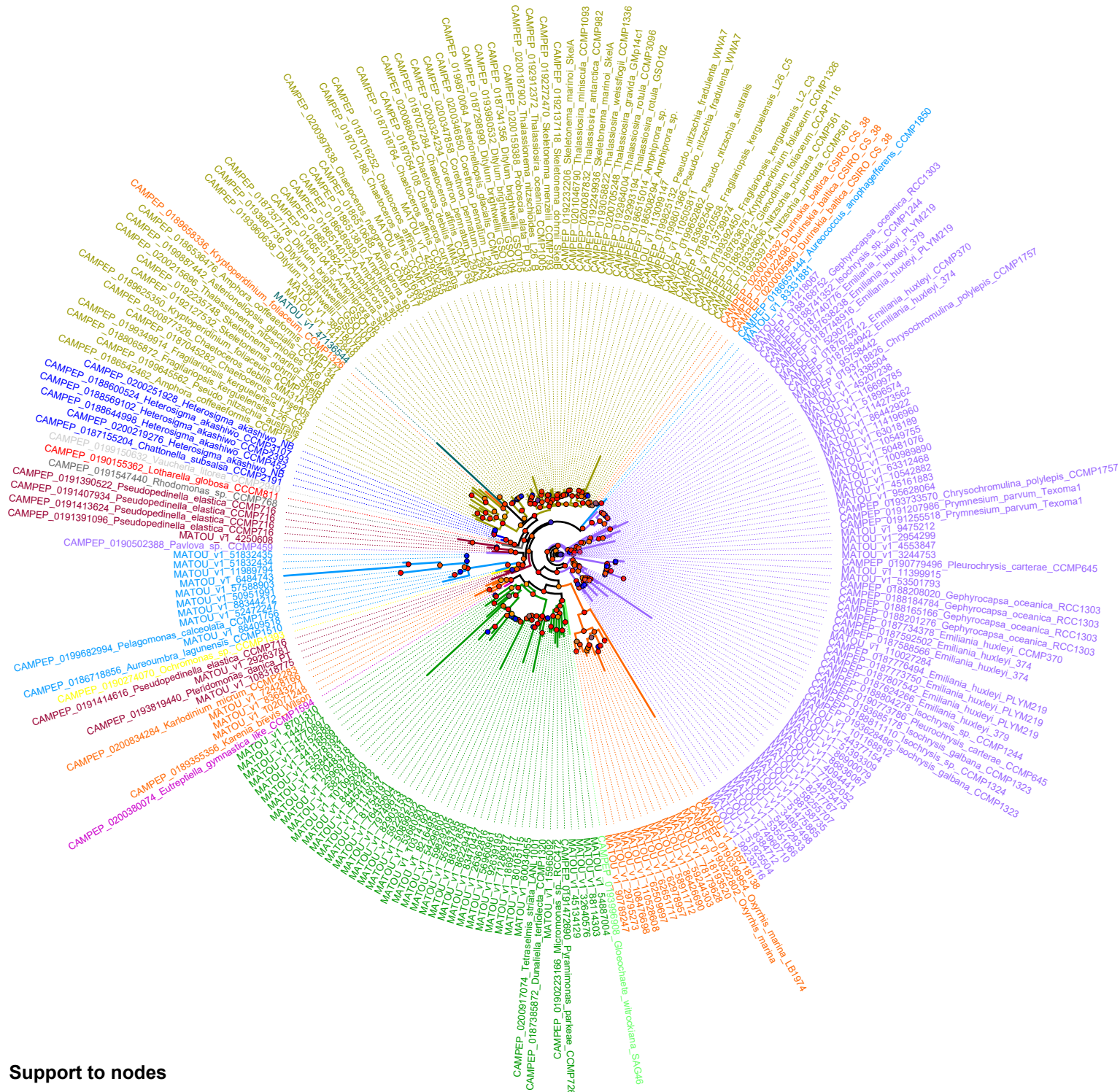

Support to nodes

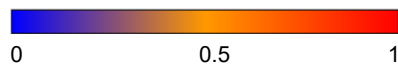

0.6

Supplement: Supplementary file 1 [file marinedrugs-18-00237-s001.zip › S3 File.pdf]
