# Supplementary material for: Monogalactosyldiacylglycerol and Sulfolipid Synthesis in Microalgae
Source: Mar Drugs. 2020 May 1;18(5):237. doi: 10.3390/md18050237 (PMC7281551; doi:10.3390/md18050237)
Supplement: Supplementary file 1 [file S2 File.pdf › S2 File.pdf]

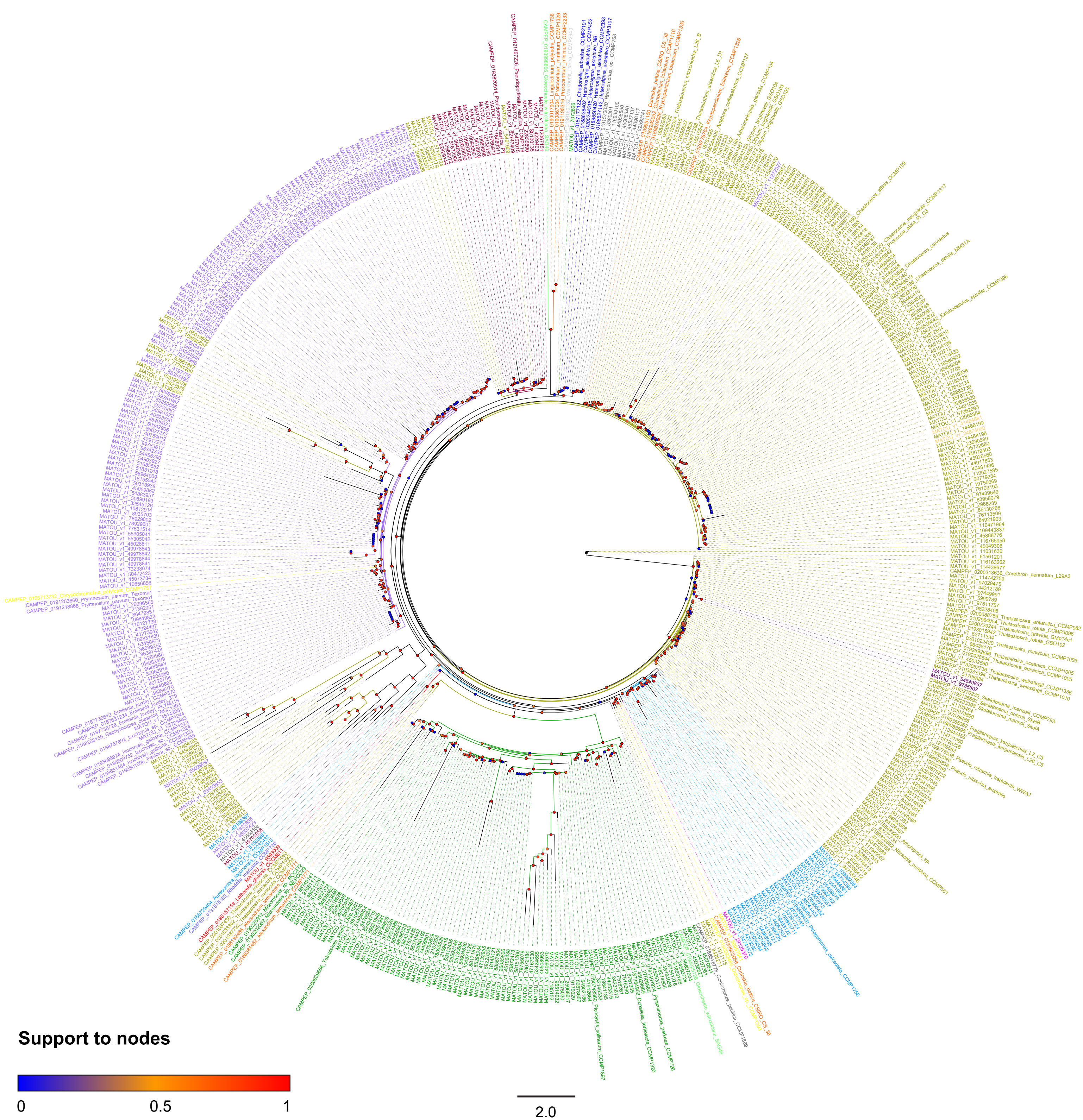
Support to nodes
0
0.5
1
2.0
